# Downregulation of Mitophagy, Complex I Biogenesis, and Signaling by ROBO Receptors—Implications for Psoriasis Pathogenesis

**DOI:** 10.3390/ijms26125546

**Published:** 2025-06-10

**Authors:** Malin Assarsson, Jan Söderman, Olaf Dienus, Oliver Seifert

**Affiliations:** 1 Department of Biomedical and Clinical Sciences, Faculty of Health Sciences, Linköping University, 581 83 Linköping, Sweden; malin.assarsson@rjl.se (M.A.);; 2 Division of Dermatology and Venereology, Region Jönköping County, 551 85 Jönköping, Sweden; 3 Laboratory Medicine, Region Jönköping County, 551 85 Jönköping, Sweden

**Keywords:** complex I biogenesis, GSEA, mitophagy, psoriasis, RNA sequencing

## Abstract

The pathogenesis of psoriasis is complex and many specific immunopathogenic mechanisms still remain unclear. Our goal was to identify novel pathways involved in the pathogenesis of psoriasis by analyzing differentially expressed genes, and to conduct pathway and cluster analysis by comparing lesional and non-lesional skin with healthy controls. Accordingly, 2 mm punch biopsies were taken from lesional elbow skin and non-affected adjacent skin of 23 patients with plaque-type psoriasis and from the elbow skin of 25 healthy controls. Differentially expressed genes were analyzed through RNA sequencing, and gene set enrichment analysis was used to analyze biological pathways. Our results showed downregulation of the pathway clusters “*Mitophagy*” and “*Respiratory Electron Transport*” when comparing both lesional and non-lesional skin to control skin. The pathway “Signaling by ROBO receptors” was downregulated in all three comparisons. Conversely, pathways relating to SUMOylation were upregulated when comparing lesional skin to both non-lesional and control skin, and those relating to the synthesis of PIPs at the early endosome membrane were found to be upregulated in lesional skin compared to control skin. The dysregulation of pathways relating to mitophagy (involved in the removal of damaged mitochondria), complex I biogenesis (a component of the mitochondrial respiratory chain), signaling by ROBO receptors (important for cell migration), and the synthesis of PIPs at the early endosome membrane (with a pivotal role in endocytic pathways and autophagy) suggests their potential role in psoriasis. Further research into the mechanisms of these dysregulated pathways, along with confirmation of protein expression levels, is necessary to validate their roles in psoriasis pathogenesis.

## 1. Introduction

Psoriasis is a chronic immune-mediated inflammatory disorder with a reported prevalence of approximately 2–3% [1,2]. It is associated with an elevated risk of severe comorbidities, including myocardial infarction and inflammatory bowel disease (IBD) [3,4,5,6,7]. The immune dysregulation in psoriasis involves excessive hyperproliferation of keratinocytes, enhanced angiogenesis, and infiltration of immune cells such as T cells, neutrophils, and macrophages into the dermis [8]. Among these, Th17 T cells play a crucial role by activating dendritic cells and keratinocytes, which in turn drive the production of antimicrobial peptides, pro-inflammatory cytokines, and chemokines [9].

The pathophysiology of psoriasis is multifaceted, including dysregulation of both the innate and adaptive immune responses. Transcriptomic studies comparing psoriatic lesional skin with healthy skin have identified differentially expressed genes (DEGs) predominantly linked to keratinization, keratinocyte differentiation, and epidermal cell maturation [10]. Cytokines and interleukins (ILs) are key contributors to disease progression [11], including tumor necrosis factor (TNF), IL-17, IL-23 nuclear factor-kappaB (NF-κB), and various chemokines [10,12,13,14], many of which are components of the JAK/STAT signaling pathway [15].

Beyond DEG analysis, gene set enrichment analysis (GSEA) has been applied to psoriasis research, revealing enriched pathways associated with IL-17 signaling, proteasome activity [16], transcription factors, JAK/STAT signaling [15], antiviral defense mechanisms [17], ribosomal function [18], vascular smooth muscle contraction, and p53 signaling [19]. Additionally, pathways related to skin development, barrier integrity, and cell cycle regulation have been identified [20].

Targeted therapies against TNF, IL-17, IL-23, and the JAK pathway have shown significant clinical efficacy in psoriasis management [21]. However, not all patients respond well to these treatments, there is no cure for the disease, and many of the specific immunopathogenic mechanisms in psoriasis still remain unclear [22]. Consequently, identification of novel pathways and pivotal genes that play essential roles in the pathophysiology of psoriasis is important to increase knowledge and to possibly find new therapeutic targets.

This study aims to explore novel biological pathways that may contribute to the development of psoriasis. Using RNA sequencing, we will analyze differentially expressed genes (DEGs) and their associated biological pathways and networks by comparing lesional and non-lesional skin of patients with psoriasis versus healthy controls.

## 2. Results

### 2.1. Differentially Expressed Genes

A total of 18,513 genes were analyzed (Supplementary Table S1). For lesional skin compared to controls, 5576 genes were significantly upregulated and 7162 genes were significantly downregulated. For non-lesional skin compared to controls, 1742 genes were significantly increased and 1186 genes were significantly decreased. For lesional skin compared to non-lesional skin, 5418 genes showed significantly increased expression and 7297 genes showed significantly decreased expression.

### 2.2. GSEA Results

**Figure 1:**
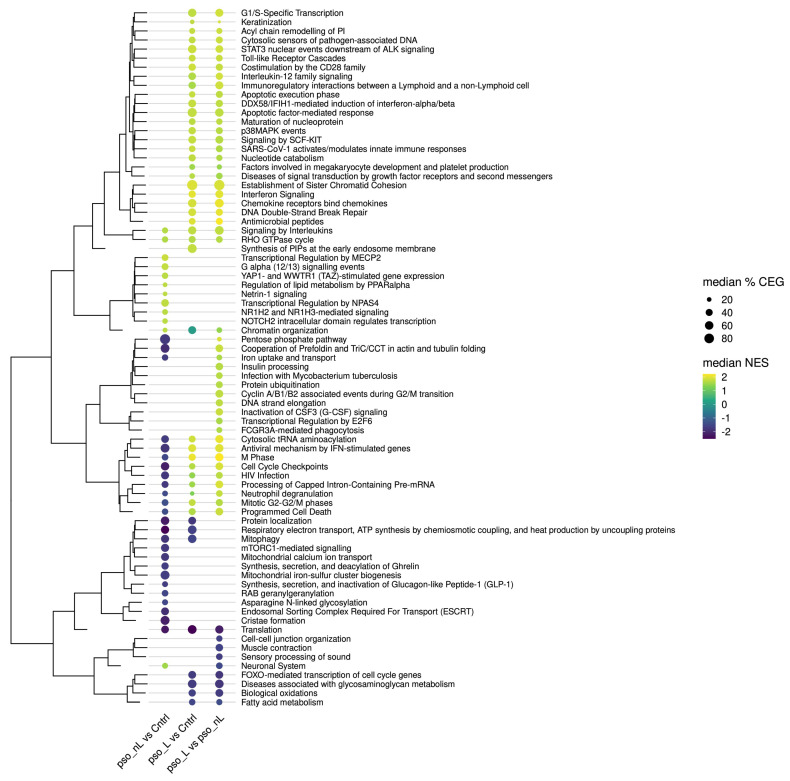
Gene set enrichment based on gene expression in biopsy samples from non-lesional (pso_NL) or lesional (pso_L) skin of psoriasis patients, or skin of healthy controls (Cntrl), visualized as a dot plot, with Reactome pathway clusters along the y-axis and pairwise group comparisons along the x-axis. For each pathway cluster, the median value of the proportions of core enriched genes (CEGs) is mapped to the dot size, and the median normalized enrichment score (NES) is mapped to the dot color, where a positive or a negative score corresponds to pathway clusters with up- or downregulated genes, respectively. Pathway clusters are further arranged (dendrogram) based on similarities in their median NES values.

**Figure 2:**
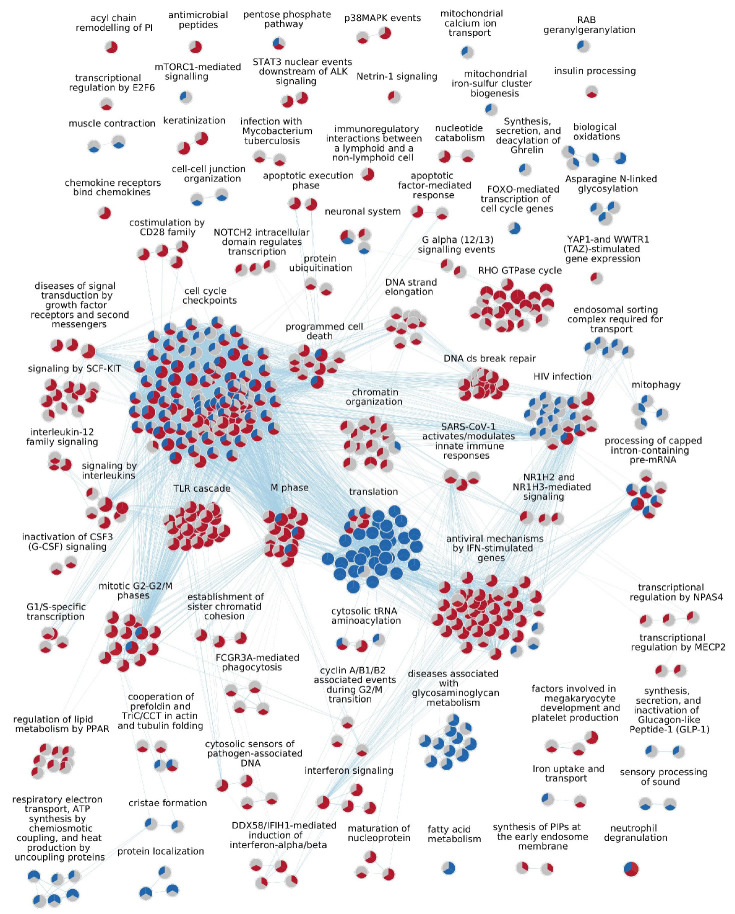
Illustration of enriched Reactome pathways based on statistical comparisons between transcriptional profiles in lesional, non-lesional, and control skin. The enrichment map shows upregulated pathways in red, downregulated pathways in blue, and pathways not significantly enriched in grey. Each node is divided into three parts, in which the left upper part represents non-lesional vs. control skin, the right upper part lesional vs. control skin, and the lower middle part lesional vs. non-lesional skin.

The GSEA and cluster analysis comparing lesional skin to control skin, lesional skin to non-lesional skin, and non-lesional skin to controls are summarized using a dot plot (Figure 1) and a node/edge network (Figure 2).

**Figure 3:**
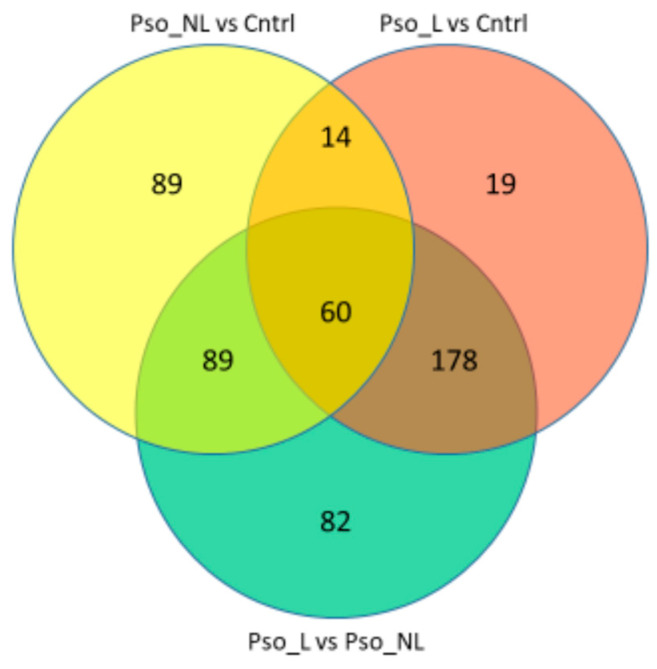
Venn diagram showing number of unique and shared regulated Reactome pathways when comparing lesional and non-lesional skin of psoriasis patients with skin of healthy controls (Pso_L = psoriasis lesional skin, Pso_NL = psoriasis non-lesional skin, Cntrl = healthy control skin).

Comparison of lesional skin to control skin identified 271 pathways, with 252 pathways for non-lesional skin vs. control skin and 409 pathways for lesional skin vs. non-lesional skin (Figure 3; Supplementary Table S2).

“*Cell cycle checkpoints*” is the largest cluster, comprising 109 pathways, which are predominantly upregulated pathways when evaluating lesional skin relative to both control and non-lesional skin. However, these pathways are primarily suppressed when assessing non-lesional skin against control skin. The second largest cluster, “*Antiviral mechanisms by IFN-stimulated genes*”, contains mainly upregulated pathways when comparing lesional skin to control and non-lesional skin, but only two downregulated pathways (“Gluconeogensis” and “HSF1 activation”) when comparing non-lesional to control skin (Figure 2).

Sixty pathways were identified in all three comparisons. The clusters “*Cell cycle checkpoints*” and “*Programmed cell death*” and the single pathway “Neutrophil degranulation” all contain several pathways that are increased when assessing lesional relative to both non-lesional and control skin; however, some pathways are suppressed when assessing non-lesional skin against control skin, such as the pathways “TCR signaling” and “Class I MHC mediated antigen processing & presentation”.

### 2.3. Upregulated Pathways

#### 2.3.1. Lesional Skin Compared to Control Skin

**Figure 4:**
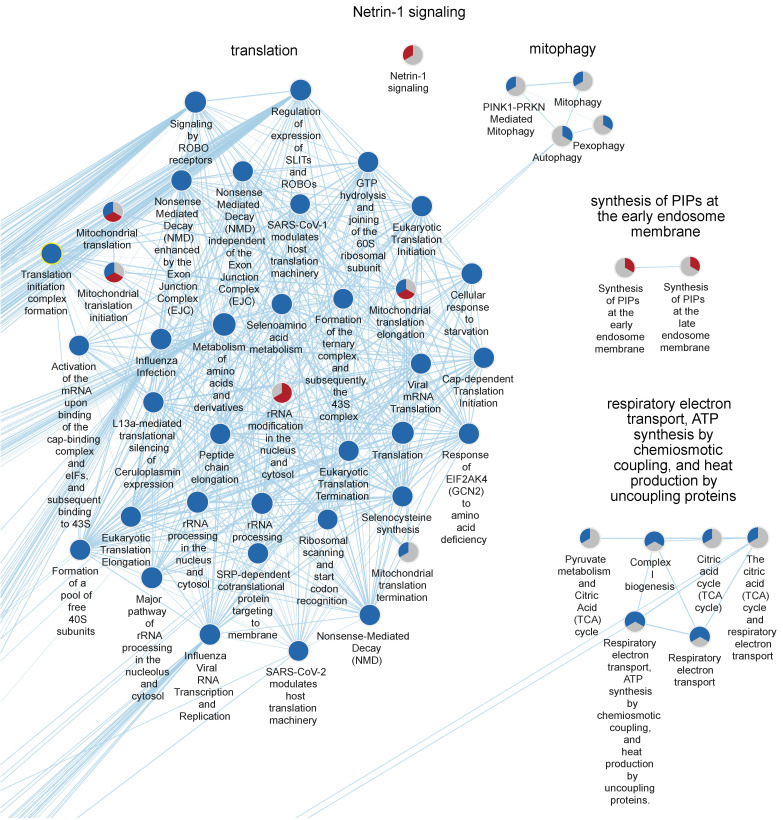
Subgraph showing key enriched Reactome pathways based on statistical comparisons between transcriptional profiles in lesional, non-lesional, and control skin. The enrichment map shows upregulated pathways in red, downregulated pathways in blue, and and pathways not significantly enriched in grey. Each node is divided into three parts, in which the left upper part represents non-lesional vs. control skin, the right upper part lesional vs. control skin, and the lower middle part lesional vs. non-lesional skin.

When comparing lesional skin to control skin, 214 pathways are significantly upregulated. The 10 most significantly upregulated pathways relate mainly to mitosis, belonging to the clusters “*M phase*” and “*Cell cycle checkpoints*” (Table 1). The other highly significant upregulated pathways belong to the clusters “*Interferon signaling*” and “*Antiviral mechanisms by IFN-stimulated genes*”. Other upregulated clusters include “*TLR cascade*” and “*Keratinization*”, along with the single pathway “Anti-microbial peptides” (Figure 1; Supplementary Table S2). A single cluster, “*Synthesis of PIPs at the early endosome membrane*”, displays two upregulated pathways unique to lesional skin compared to control samples (Figure 1 and Figure 4)

#### 2.3.2. Non-Lesional Skin Compared to Control Skin

When comparing non-lesional to control skin, 53 pathways are significantly upregulated (Figure 2; Supplementary Table S2). The 10 most significantly upregulated pathways when assessing non-lesional skin against control skin have four pathways belonging to the cluster “*Chromatin organization*” and three pathways belonging to the cluster “*RHO GTPase cycle*” (Table 1). Several upregulated pathways are unique to the comparison, including the cluster “*Transcriptional regulation by NPAS4*” and the single pathway “Netrin-1 signaling” (Figure 1 and Figure 4; Supplementary Table S2).

#### 2.3.3. Lesional Skin Compared to Non-Lesional Skin

When comparing lesional skin to non-lesional skin, 355 pathways are significantly upregulated, which is the highest number of pathways among the comparisons. Among the 10 most significantly upregulated pathways, three pathways belong to the cluster “*Chromatin organization*” and two to the cluster “*M phase*” (Table 1). Lesional skin compared to non-lesional skin has upregulated clusters in common with lesional skin compared to control skin, including “*TLR cascade*” and “*Keratinization*”, along with the single pathway “Anti-microbial peptides” (Figure 1; Supplementary Table S2). The cluster “*Apoptotic factor-mediated response*”, containing two pathways, is upregulated. One of the pathways, “Cytochrome c-mediated apoptotic response”, is also upregulated when comparing lesional skin to control skin, but neither is regulated when comparing non-lesional to lesional skin. In general, lesional skin compared to non-lesional skin has significantly more upregulated pathways than non-lesional skin compared to control skin (Figure 2, Supplementary Table S2).

### 2.4. Downregulated Pathways

#### 2.4.1. Lesional Skin Compared to Control Skin

When comparing lesional skin to control skin, 57 pathways are downregulated. All of the 10 most significantly downregulated pathways belong to the cluster “*Translation*” (Table 1). Other downregulated clusters include “*Biological oxidations*”, “*Mitophagy*”, and “*Respiratory electron transport*” (Figure 1 and Figure 4).

#### 2.4.2. Non-Lesional Skin Compared to Control Skin

When comparing non-lesional skin to control skin, 199 pathways are significantly downregulated, which is the highest number among the three comparisons. Of the 10 most significantly downregulated pathways, seven belong to the cluster “*Translation*” (Table 1). Several clusters, for example, “*Programmed cell death*” and “*HIV infection*”, are downregulated when comparing non-lesional to control skin, but upregulated when comparing lesional to both control and non-lesional skin (Figure 1; Supplementary Table S2). The cluster “*Mitophagy*” is downregulated both when comparing non-lesional skin to control skin and lesional skin to control skin. Several other pathways relating to mitochondria are downregulated when comparing non-lesional to control skin. Other downregulated pathways unique to the comparison of non-lesional skin to control skin include the cluster “*Endosomal sorting complex required for transport*” and the single pathway “RAB geranylgeranylation”.

#### 2.4.3. Lesional Skin Compared to Non-Lesional Skin

When comparing lesional to non-lesional skin, 54 pathways are significantly downregulated. Of the 10 most significantly downregulated pathways, all ten belong to the cluster *Translation* (Table 1). Other downregulated clusters include “*Diseases associated with glycosaminoglycan metabolism*” and “*Biological oxidations*”, which are also downregulated when comparing lesional skin to control skin (Figure 1; Supplementary Table S2).

## 3. Discussion

In addition to confirming the enrichment of well-known pathways, our GSEA results reveal several less-known affected biological pathways that have not been previously described in the pathogenesis of psoriasis.

The cluster *Mitophagy* encompassed suppressed pathways, including “Autophagy” and “Pexophagy”, in lesional skin when assessed against controls, as well as the pathway “Mitophagy”, which was suppressed in non-lesional skin relative to controls. Autophagy is a fundamental cellular process responsible for degrading and recycling intracellular components to sustain homeostasis, exerting cytoprotective and anti-inflammatory effects [23]. Mitophagy, a specialized form of autophagy, facilitates the selective degradation of damaged mitochondria [24]. Compromised autophagy leads to the accumulation of dysfunctional organelles, contributing to dysregulated antigen presentation and immune cell activation, which can drive chronic inflammatory conditions [25]. Genetic associations between autophagy-related loci and inflammatory diseases, including rheumatoid arthritis and inflammatory bowel disease, have been proposed [23]. In psoriasis, autophagy has been implicated in disease pathogenesis, specifically through polymorphisms in the ATG16L1 gene [26,27,28], which also have a significant role in epidermal keratinization [29]. We found the expression of the gene ATG16L to be significantly suppressed and that of the genes ATG3 and ATG4C to be significantly higher in the lesional skin of psoriasis patients compared to controls and to non-lesional skin.

Autophagy inhibition has been shown to modulate IL-23A secretion in innate immune cells, including dendritic cells [30]. Additionally, IL-17a, important in the pathophysiology of psoriasis, has been found to suppress autophagy by preventing autophagosome development [31]. Several treatments for psoriasis, such as UVB phototherapy, retinoids, and JAK inhibitors, have been reported to stimulate autophagy [32,33,34]. These results suggest that modifying autophagy might be an interesting future therapeutic avenue for psoriasis research.

The cluster “*Respiratory electron transport, ATP synthesis by chemiosmotic coupling, and heat production by uncoupling proteins*” comprises three suppressed pathways in lesional skin relative to controls, one of which is “Complex I Biogenesis”. Complex I is responsible for establishing a proton gradient across the mitochondrial inner membrane to drive ATP production [35]. Genetic variations in mitochondrial complex I have been connected to several different diseases including cancer [36,37,38], and inhibitors aiming at complex I are evolving as promising therapeutics [39]. Pathway enrichment linking to the mitochondrial respiratory chain has previously been reported in ulcerative colitis and Crohn’s disease [40,41]. A small study recognized NDUFB7, a complex I subunit, as a promising biomarker of psoriatic arthritis [42]. However, to our knowledge, altered gene expression correlated to complex I biogenesis has not been previously described in psoriasis.

The downregulation of “Complex I biogenesis” is particularly intriguing in conjunction with the suppression of “Mitophagy”. Elevated intracellular reactive oxygen species (ROS) levels can result from mitochondrial dysfunction, and impaired autophagy can result in the accretion of dysfunctional mitochondria, leading to oxidative stress and inflammation [43,44]. This interplay between reduced autophagy and impaired complex I function may contribute to heightened inflammation in psoriasis. Remarkably, several mitochondria-related pathways were suppressed exclusively in non-lesional skin relative to controls, suggesting that mitochondrial dysfunction may extend beyond lesional areas, potentially playing a role in the broader pathophysiology of psoriasis.

The “Signaling by ROBO receptors” pathway within the *Translation* cluster is significantly suppressed across all three comparisons, a finding not hitherto reported in psoriasis. Initially recognized for its role in axon guidance, the “Slit glycoprotein and Roundabout (Slit/Robo)” signaling pathway has also been associated with various cancers [45,46] and immune regulation, particularly through its influence on dendritic cell migration [45]. Given that angiogenesis and immune response are central to psoriasis pathophysiology [1,47], the involvement of Slit/Robo signaling in these processes is noteworthy. Emerging evidence proposes that the Slit/Robo pathway may regulate WNT/Beta-catenin signaling [48], which has been extensively linked to psoriasis development [49,50,51]. Abnormalities in the peripheral nervous system are believed to be of importance in the pathogenesis of psoriasis, and differences in gene expression of genes relating to neuritogenesis, including Slits, have been found when comparing lesional to non-lesional skin of psoriasis patients [52]. The authors suggest that these alterations can affect T-cell activation and infiltration through crosstalk in the neuroimmune system [52]. This further strengthens the potential connection between the Slit/Robo signaling pathway and psoriasis pathophysiology.

Intriguingly, non-lesional skin displays distinct pathway regulation not observed in either lesional or control areas. Notably, several increased pathways are connected to neuronal migration and axon guidance, such as Netrin-1, as well as being linked to key neurobiological processes, such as NPAS4 [53,54]. While NPAS4’s direct implication in skin biology remains unclear, insights from neuroimmunology suggest a potential connection between neuronal activity and skin health. On the other hand, suppressed pathways in non-lesional skin encompass endosomal sorting and vesicle trafficking regulation [55,56]. These findings prompt speculation about the unique regulatory landscape of non-lesional skin.

A distinct cluster, *synthesis of PIPs at the early endosome membrane*, revealed two increased pathways specific to lesional skin relative to control samples. The “phosphatidylinositol 3,5-bisphosphate (PI(3,5)P2)” pathway, a key component of this cluster, is vital for endocytic pathways, membrane trafficking, autophagy, and stress adaption. Mutations that impair PI(3,5)P2 biosynthesis are related to various human diseases including neurodegenerative disorders [57]. Although the particular role of endosome membrane dynamics and PI(3,5)P2 signaling in psoriasis pathophysiology is not yet fully understood, our results suggest that modifications in these cellular processes may potentially contribute to the inflammatory aspects of psoriasis.

Alongside these newly identified pathways, our results corroborate previous studies using GSEA. We observed similar enrichments in pathways relating to the cell cycle, interferon signaling, antiviral mechanisms, TCR signaling, and ribosomes [17,18,20].

Our study has several strengths. The consistency in the location of skin samples minimizes the likelihood that the observed differences are due to regional variations. By adjusting for age and gender, we control for potential confounding factors, enabling more precise comparisons between patient and control groups. However, the relatively small number of patients included may limit the generalizability of our conclusions and affect the statistical power of our study. A larger sample size would yield more robust results, potentially capturing more subtle differences between groups. Another limitation is that our results are based solely on RNA sequencing data and need to be verified through analysis of protein expression or functional experiments. Additionally, accounting for other potential confounding factors such as disease severity, ongoing medications, or other illnesses could further support the study’s conclusions.

In conclusion, our GSEA results highlight several novel biological pathways not previously described in the development of psoriasis. Further research with larger sample sizes and protein expression level analyses is necessary to validate these findings and explore these pathways as potential therapeutic targets for the disease.

## 4. Materials and Methods

### 4.1. Study Subjects

This study included 50 individuals diagnosed with plaque psoriasis and 77 healthy control subjects. To ensure the reliability of the findings, participants had not taken oral antibiotics, anti-inflammatory medications, or immune-modulating treatments for at least three months before enrollment and throughout the study period. Individuals were excluded if they were pregnant, under 18 years of age, diagnosed with malign disorders, psoriatic arthritis, or systemic inflammatory diseases, or had active infections at the time of sampling.

All participants provided written informed consent in accordance with protocols approved by the Ethical Committee of Linköping University, Linköping, Sweden (Institutional Review Board approval number 2014/179-31). Data on gender, age, ongoing psoriasis treatments, and current medications were collected. Disease severity was assessed by a trained dermatology nurse using the Psoriasis Area and Severity Index (PASI) [58].

To standardize sampling conditions, participants refrained from applying anti-inflammatory ointments on the skin two weeks before sample collection. Topical emollients were permitted. A subset of 23 psoriasis patients and 25 healthy controls was selected for RNA sequencing, as described in Section 4.3. The demographic characteristics of the study population are summarized in Table 2.

### 4.2. Sample Collection and Skin Biopsy Procedure

Punch biopsies, 2 mm in diameter, were attained from the lesional skin of the elbow and from the non-lesional skin neighboring the affected elbow from patients with psoriasis. In healthy controls, samples were taken from the elbow region. To preserve RNA integrity, the biopsies were instantaneously placed in RNAlater (Qiagen, Hilden, Germany) and afterwards kept at −80 °C.

### 4.3. RNA Extraction and Sequencing

The skin samples were processed with a TissueRuptur and disposable probes (Qiagen, Hilden, Germany) to achieve homogenization. Total RNA was isolated using the RNeasy Fibrous Tissue mini kit (Qiagen, Hilden, Germany). RNA concentrations were measured with the Qubit 2.0 Fluorometer, employing the Qubit RNA BR assay (Thermo Fisher Scientific, Waltham, MA, USA). RNA quality was assessed using the Agilent 2100 Bioanalyzer and RNA 6000 Nano kit (Agilent Technologies, Santa Clara, CA, USA), with samples displaying an RNA integrity number (RIN) below 7.7 being omitted. Accordingly, RNA sequencing was carried out with 23 psoriasis patients and 25 healthy controls. Poly(A)-enriched RNA was isolated, and mRNA libraries were prepared using the TruSeq Stranded mRNA (Illumina, San Diego, CA, USA). Libraries were then clustered on cBot, and sequencing was conducted with HiSeq SBS v4 chemistry and a HiSeq 2500 system (Illumina, San Diego, CA, USA) with a read length of 1 × 50 nucleotides (single-read setup). Sequencing depth ranged from 20.5 to 35.9 million reads per sample, with an average of 28.8 million reads.

### 4.4. Bioinformatic Processing and Statistical Analysis

RNA sequencing data were processed and examined with R version 4.2.2 (version 2022.12.0; https://posit.co/), utilizing packages from the Bioconductor release 3.16 (https://bioconductor.org/) unless otherwise stated.

For all statistical analyses, unless otherwise specified, the false discovery rate (FDR) was controlled by applying the Benjamini–Hochberg correction, and a significance threshold of adjusted *p*-values < 0.05 was used.

The sequencing reads were aligned to the primary GRCh38 reference genome from Ensembl and mapped to the corresponding genetic features (official gene symbols) based on the latest genomic annotation file (version 111). The feature counts were generated at gene rank by applying the RSubread package. The alignment efficacy ranged from 97.5% to 98.6%, with a mean of 98.3%. This provided a read depth of 20.1–35.3 million aligned reads per sample, with a mean of 28.3 million reads. Genes showing more than 1 count per million in at least as many samples as the smallest set (i.e., 23) were taken for further investigation. Gene annotations, including their approved complete names, Entrez gene IDs, and chromosomal positions, were retrieved by applying the AnnotationDbi and org.Hs.eg.db packages. Genes with inadequate data were disqualified from subsequent investigation.

Normalization of gene expression data was performed by scaling factors derived from the trimmed mean of the M-values, implemented via the calcNormFactors function in the edgeR package. Differential expression (DE) analysis was carried out with the limma package. To address the within-subject comparisons (e.g., lesional vs. non-lesional skin in psoriasis patients) and between-subject comparisons (e.g., psoriatic skin vs. control skin), the voomLmFit function was applied, which is similar to calling voom, followed by duplicateCorrelation and lmFit. Skin type was investigated for DE genes, while adjusting for gender and age, by an empirical Bayes moderated *t*-test. Significance testing for every single gene was performed to assess alterations between groups with respect to zero.

### 4.5. Gene Set Analysis, Network Analysis, and Visualization

Gene set enrichment analysis (GSEA), network exploration, and visualization were achieved following the methods outlined in a previous study [40]. In summary, Reactome pathways (https://reactome.org/), were evaluated using pre-ranked gene lists, with ranking established on fold change and unadjusted *p*-values as of the empirical Bayes moderated *t*-test, facilitated by the ReactomePA package. Pathways with an adjusted *p*-value < 0.05 were selected for clustering analysis with Cytoscape v3.9.1, combined with the EnrichmentMap v3.3.4 and clusterMaker2 v2.2 plugins. The cluster associated with the pathway exhibiting the lowest adjusted *p*-value was assigned as the cluster’s name. For visualization, a dot plot was generated, displaying all clusters, which were further grouped hierarchically according to their median normalized enrichment score (NES) values. The dimensions and coloring of the dots in the plot indicate the median percentage of core enriched genes (CEGs), contributing to the enrichment score, and the median NES values, respectively.

## Figures and Tables

**Table 1:** Reactome enrichment analysis of the differentially expressed genes. Selection of the most significant clusters (NES = normalized enrichment score, CEGs = core enriched genes, Comb. FDR Bonferroni = combined false discovery rate *p*-values using Bonferroni method).

Cluster Name	Median NES	Median % CEGs	Comb. FDR Bonferroni
**UPREGULATED**			
**lesional vs. control**			
M phase	2.07	42	6.6 × 10^−13^
Interferon signaling	2.02	42.5	5.3 × 10^−11^
Cell cycle checkpoints	1.65	33	1.7 × 10^−9^
Signaling by interleukins	1.73	55	9.9 × 10^−7^
Antiviral mechanism by IFN-stimulated genes	1.96	50	1.8 × 10^−6^
**non-lesional vs. control**			
Chromatin organization	1.69	21	4.4 × 10^−5^
RHO GTPase cycle	1.63	32	2.3 × 10^−4^
G alpha (12/13) signaling events	1.76	38	1.0 × 10^−3^
Transcriptional regulation by NPAS4	1.81	37	2.3 × 10^−3^
Netrin-1 signaling	1.72	20	8.1 × 10^−3^
**lesional vs. non-lesional**			
Antiviral mechanism by IFN-stimulated genes	1.98	56	7.4 × 10^−7^
Keratinization	1.92	16	3.7 × 10^−6^
HIV infection	1.70	38	6.1 × 10^−6^
Antimicrobial peptides	2.20	39	1.7 × 10^−5^
DNA double-strand break repair	2.04	41	3.1 × 10^−5^
**DOWNREGULATED**			
**lesional vs. control**			
Translation	−2.30	64	3.8 × 10^−21^
Protein localization	−1.78	49.5	2.5 × 10^−3^
Fatty acid metabolism	−1.53	33	8.0 × 10^−3^
Biological oxidations	−1.63	43	8.6 × 10^−3^
Diseases associated with glycosaminoglycan metabolism	−1.72	62	1.2 × 10^−2^
**non-lesional vs. control**			
Translation	−2.30	48	9.4 × 10^−27^
Respiratory electron transport, ATP synthesis by chemiosmotic coupling, and heat production by uncoupling proteins	−2.52	55	6.1 × 10^−19^
Protein localization	−2.22	59	2.9 × 10^−9^
Cell cycle checkpoints	−2.19	58	3.9 × 10^−7^
Processing of capped intron-containing pre-mRNA	−1.79	39	1.8 × 10^−6^
**lesional vs. non-lesional**			
Translation	−2.18	55	3.7 × 10^−15^
Diseases associated with glycosaminoglycan metabolism	−1.81	61	8.9 × 10^−3^
Sensory processing of sound	−1.69	35	9.1 × 10^−3^
F cell cycle genes	−1.79	50	1.0 × 10^−2^
Muscle contraction	−1.59	46	1.2 × 10^−2^

**Table 2:** Patients’ demographic characteristics.

	Psoriasis	Control
Number of subjects	23	25
Age, years, mean ± SD ^a^ (range)	53.7 ± 14.3 (22–71)	47.0 ± 18.1 (19–86)
Male/female ratio, n	12:11	11:14
PASI, mean ± SD (range)	5.3 ± 3.3 (0.5–11.3)	-
Family history of psoriasis, %	69.6	0

^a^ Standard deviation.

## Data Availability

The data that support the findings of this study are available from the corresponding author, O.S., upon reasonable request.

## Supplementary Materials

The following supporting information can be downloaded at https://www.mdpi.com/article/10.3390/ijms26125546/s1.
